# Temporal and spatial expression of genes involved in DNA methylation during reproductive development of sexual and apomictic *Eragrostis curvula*

**DOI:** 10.1038/s41598-017-14898-5

**Published:** 2017-11-08

**Authors:** J. P. Selva, L. Siena, J. M. Rodrigo, I. Garbus, D. Zappacosta, J. R. Romero, J. P. A. Ortiz, S. C. Pessino, O. Leblanc, V. Echenique

**Affiliations:** 1 CERZOS-CONICET, CCT-Bahía Blanca, 8000 Bahía Blanca, Argentina; 2 Departamento de Biología, Bioquímica y Farmacia, Universidad Nacional del Sur, 8000 Bahía Blanca, Argentina; 3 IICAR-CONICET/Laboratorio de Biología Molecular, Facultad de Ciencias Agrarias, Universidad Nacional de Rosario, Parque Villarino, S2125ZAA Zavalla, Argentina; 4 Departamento de Agronomía, Universidad Nacional del Sur, 8000 Bahía Blanca, Argentina; 5 Departamento de Ciencias de la Salud, Universidad Nacional del Sur, 8000 Bahía Blanca, Argentina; 6 DIADE, IRD, Univ Montpellier, Montpellier, France

## Abstract

Recent reports in model plant species have highlighted a role for DNA methylation pathways in the regulation of the somatic-to-reproductive transition in the ovule, suggesting that apomixis (asexual reproduction through seeds) likely relies on RdDM downregulation. Our aim was therefore to explore this hypothesis by characterizing genes involved in DNA methylation in the apomictic grass *Eragrostis curvula*. We explored floral transcriptomes to identify homologs of three candidate genes, for which mutations in *Arabidopsis* and maize mimic apomixis (*AtAGO9/ZmAGO104*, *AtCMT3/ZmDMT102/ZmDMT105*, and *AtDDM1/ZmCHR106*), and compared both their spatial and temporal expression patterns during reproduction in sexual and apomictic genotypes. Quantitative expression analyses revealed contrasting expression patterns for the three genes in apomictic vs sexual plants. *In situ* hybridization corroborated these results for two candidates, *EcAGO104* and *EcDMT102*, and revealed an unexpected ectopic pattern for the *AGO* gene during germ line differentiation in apomicts. Although our data partially support previous results obtained in sexual plant models, they suggest that rather than an RdDM breakdown in the ovule, altered localization of *AtAGO9/ZmAGO104* expression is required for achieving diplospory in *E*. *curvula*. The differences in the RdDM machinery acquired during plant evolution might have promoted the emergence of the numerous apomictic paths observed in plants.

## Introduction

Unlike the genetically diverse progeny produced by sexual reproduction in flowering plants, the asexual reproductive process of apomixis results in seeds containing maternal clones^1^. Apomixis is widely distributed among angiosperms^2^, and its emergence is thought to have required a deregulation of both the genetic and epigenetic components of female reproduction in sexual ancestors^3^.

Seed formation in sexual plants is achieved through a double fertilization event after the encounter of the female gametophyte with the male gametophyte, both carrying two gametes. Gametophytes typically differentiate from meiotic products, the spores. Female meiosis initiates within a single cell, specified in the nucellus of the ovule, the megaspore mother cell (MMC). Upon meiosis the MMC produces a tetrad of reduced cells, out of which a single functional product, the megaspore (MS), undergoes mitotic divisions and differentiation. The resulting gametophyte, or the embryo sac (ES), is seven-celled at maturity and contains two female gametes, the egg cell and the binucleate central cell^4^. During the double fertilization process, each female gamete fuses with one of the two sperm cells delivered by the pollen grain (the male gametophyte). This triggers the formation of the embryo and the endosperm, respectively, and initiates seed development with the two fertilization products embedded in maternal tissues.

Apomixis refers to alternative reproductive pathways that allow somatic (i.e., unreduced) cells within the ovule to acquire a reproductive fate after the triggering of either embryogenesis (adventitious embryony) or female gametogenesis in the absence of meiosis followed by autonomous egg cell development (gametophytic apomixis)^5^. Gametophytic apomixis involves a broad spectrum of developmental pathways and is sub-categorized into either (1) diplospory, where the unreduced ES, derives from a MMC that fails to achieve meiosis and initiates gametogenesis, or (2) apospory, when several nucellar or integumental cells differentiate directly into unreduced ESs. In all gametophytic apomicts, seed development requires endosperm formation either autonomously or after central cell fertilization (pseudogamy)^5^.

Gametophytic apomixis is controlled genetically by a few undetermined factors^6^. Recent functional studies highlighted a role for small interfering RNAs (siRNAs) in apomictic pathways, as the depletion of an RNA-directed DNA methylation (RdDM) pathway specifically acting in plant ovules promotes an asexual reproductive fate^3^. This observation is supported by transcriptomic analyses in several species, e.g. *Boechera gunnisoniana* ^7^, *Hypericum perforatum* ^8,9^ and *Hieracium* spp.^10^. Members of the *ARGONAUTE4* clade in *Arabidopsis thaliana* (*AGO4*, *AGO6*, *AGO8* and *AGO9*) encode proteins binding heterochromatic siRNAs that target sequence repeats and transposable elements, and mutations in these genes result in the formation of extra female gametophytic precursors, suggesting they play a role in the somatic-to-reproductive transition in ovules^11,12^. In maize (*Zea mays*), loss-of-function of *AGO104*, a homolog of *Arabidopsis AGO9*, results in functional unreduced female gametes arising from mitosis-like divisions in the MMC, but without affecting the number of germ cells^13^. *AtAGO9* and *ZmAGO104* therefore seem to promote different functions during reproduction in *Arabidopsis* and maize, respectively repressing the germ cell fate of somatic cells and the somatic cell fate in the germ line^3^. This difference likely explains the phenotypic consequences of *AtAGO9*/*ZmAGO104* depletion, which mimics apospory in *Arabidopsis* and diplospory in maize.

It was reported that after the deregulation of several other genes involved in DNA methylation pathways apomixis-like phenotypes were observed in model plant species^11,14^. These genes include: 1) the sequences encoding two *Arabidopsis* DNA methyltransferases (DMTs) *CHROMOMETHYLTRANSFERASE3* (*CMT3*) and *DOMAIN REARRANGED METHYLTRANSFERASE1* (*DRM1*), as well as their maize respective homologs *ZmDMT102/ZmDMT105* and *ZmDMT103*; 2) the *Arabidopsis DECREASE IN DNA METHYLATION1* (*DDM1*) chromatin-remodeling factor and its maize homolog *ZmCHR106;* and 3) the *Arabidopsis* sequences regulating small interfering RNAs biogenesis *RNA-DEPENDENT RNA POLYMERASE2* (*RDR2*), *DICER-LIKE3* (*DCL3*) and *DNA-DIRECTED RNA POLYMERASES V* and *IV*. Other than RT-PCR analyses in diplosporous wild relatives of *Arabidopsis* and maize, (*Boechera holboellii* and *Tripsacum dactyloides*, respectively)^14^, no studies have investigated the role of specific epigenetic pathways in natural apomicts.

*Eragrostis curvula* (weeping lovegrass) is a perennial grass that reproduces mainly by diplosporous apomixis, although fully sexual plants occur sporadically^15,16^. The use of this model system allowed us to test for a link between the deregulation of DNA methylation pathways and the occurrence of apomixis in a natural grass system. To this end, we identified the homologs of *AtAGO9/ZmAGO104*, *ZmDMT102* and *ZmCHR106* using *E*. *curvula* floral transcriptomes and showed they were differentially expressed in the ovules of sexual and apomictic plants. Our results support the findings of previous functional analyses, performed mainly in sexual model plant species, for an essential role of the RdDM pathway in the switch from sexual to diplosporous development.

## Results

### Transcript identification in sexual and apomictic *E*. *curvula* transcriptome databases

Recently we reported an *E*. *curvula* reference transcriptome^17^ that constitutes an important step toward the identification of genes controlling key steps of the apomictic pathway. Roche 454 sequencing technology was used to generate reads from inflorescences of *E*. *curvula* apomictic and sexual genotypes. The resulting reads were *de novo* assembled using the Newbler Assembler software v2.6 (Roche, Indianapolis, IN, USA) into 49568 isotigs that were further grouped into 25186 isogroups. Near 90% of the unigenes showed high similarity to sequences from public databases.

To identify the *E*.*curvula* orthologs of the three candidate genes we selected, protein sequences of the maize AGO^18^, DMT^19^, and CHR^14^ families were queried against the *E*. *curvula* reference transcriptome. High levels of sequence similarity were found for most queries, including 11 out of 17 AGOs, five of the eight DMTs, and all eight CHR members (Tables 1, 2 and 3, respectively).

**Table 1:** Members of the ARGONAUTE family queried against the *Eragrostis curvula* protein prediction databases.

Query^a^	Subject (*E. curvula*)	% identity	Alignment length	e-value	Score
ZmAGO1a	N/A	N/A	N/A	N/A	N/A
ZmAGO1b	isotig10363	93.98	1079	0	2047
	isotig09228	87.81	1124	0	1949
ZmAGO1c	N/A	N/A	N/A	N/A	N/A
ZmAGO1f	isotig33848	84.75	1056	0	1755
	isotig18801	78.36	1049	0	1593
ZmAGO2a	N/A	N/A	N/A	N/A	N/A
ZmAGO2b	isotig33909	72.96	858	0	1228
ZmAGO4	isotig12569	87.06	904	0	1598
	isotig25978	61.94	867	0	1061
ZmAGO5a	isotig03447	70.91	495	0	699
ZmAGO5b	isotig18741	81.56	526	0	869
ZmAGO5c	isotig25155	86.95	659	0	1145
ZmAGO5d	N/A	N/A	N/A	N/A	N/A
ZmAGO7	isotig38102	86.17	412	0	739
ZmAGO9	isotig33988	86.51	912	0	1622
ZmAGO10a	N/A	N/A	N/A	N/A	N/A
ZmAGO10b	isotig33998	96.23	664	0	1316
ZmAGO18a	isotig34174	68.44	716	0	968
ZmAGO18b	N/A	N/A	N/A	N/A	N/A

^a^Query names as described by^18^. They renamed ZmAGO104 to ZmAGO9; therefore, the isotig33988 is the ortholog of ZmAGO104.

**Table 2:** Members of the DNA methyltransferase family queried against the *Eragrostis curvula* protein prediction databases.

Query^a^	Subject (*E. curvula*)	% identity	Alignment length	e-value	Score
ZmMET1a	isotig33722	87.61	702	0	1289
	isotig33939	70.82	682	0	1026
ZmMET1b	N/A	N/A	N/A	N/A	N/A
ZmMET2a (ZmDMT102/105)	isotig25116	69.84	610	0	866
ZmMET2b	N/A	N/A	N/A	N/A	N/A
ZmMET3a	isotig22920	81.68	382	0	662
ZmMET3b	N/A	N/A	N/A	N/A	N/A
ZmMET3c	isotig25609	72.21	457	0	642
ZmMET4	isotig27287	84.64	358	0	619

^a^Query names as described by^19^. They renamed ZmDMT102 as ZmMET2a; therefore, isotig25117 is the ortholog of ZmDMT102.

**Table 3:** Members of the SNF2 super family queried against the *Eragrostis curvula* protein prediction databases.

Query^a^	Subject (*E. curvula*)	% identity	Alignment length	e-value	Score
ZmCHR125	isotig16268	85.32	1260	0	2145
ZmCHR113	isotig13154	81.61	1338	0	2186
ZmCHR120	isotig08900	63.36	1261	0	1474
ZmCHR126	isotig02865	93.11	1118	0	2051
ZmCHR106	isotig25194	85.59	708	0	1204
ZmCHR101	isotig25194	84.97	712	0	1211
ZmCHR112	isotig34902	61.25	542	0	679
	isotig37943	81.98	394	0	660
ZmCHR110	isotig02865	93	928	0	1718

^a^Query names as described by^14^.

**Figure 1:**
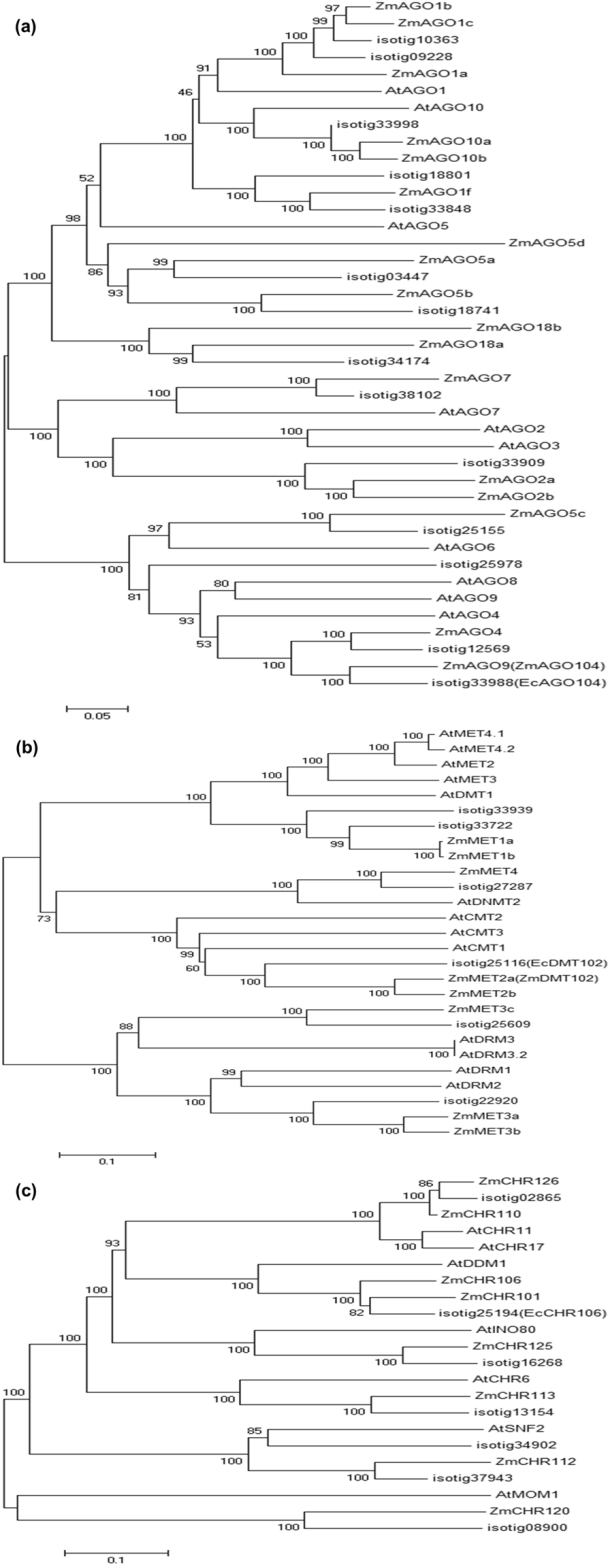
Phylogenetic trees of *E*. *curvula* RNA-dependent DNA methylation proteins in *A*. *thaliana* and *Z*. *mays*. (**a**) AGO, (**b**) DMT, and (**c**) CHR proteins from *Arabidopsis* and maize were retrieved from the The Arabidopsis Information Resource (TAIR) and the Maize Genetic and Genomic Database (MaizeGDB), respectively. The amino acid sequences of *E*. *curvula* were obtained from our floral reference transcriptomes. The unrooted neighbor-joining tree was constructed using MEGA v6.0.

To support these results, we next generated maximum likelihood phylogenetic trees for the AGO, DMT, and CHR families using protein sequences from maize, *Arabidopsis* and *Eragrostis*. As shown in Fig. 1, the clustering of homologs confirmed relatedness between *Eragrostis* sequences and that whose loss of function leads to reproductive behaviors reminiscent of apomixis in *Arabidopsis* and maize. Thus, we renamed isotig33988 as *EcAGO104*, isotig25116 as *EcDMT102* and isotig25194 as *EcCHR106*.

### Quantitative expression analysis

**Figure 2:**
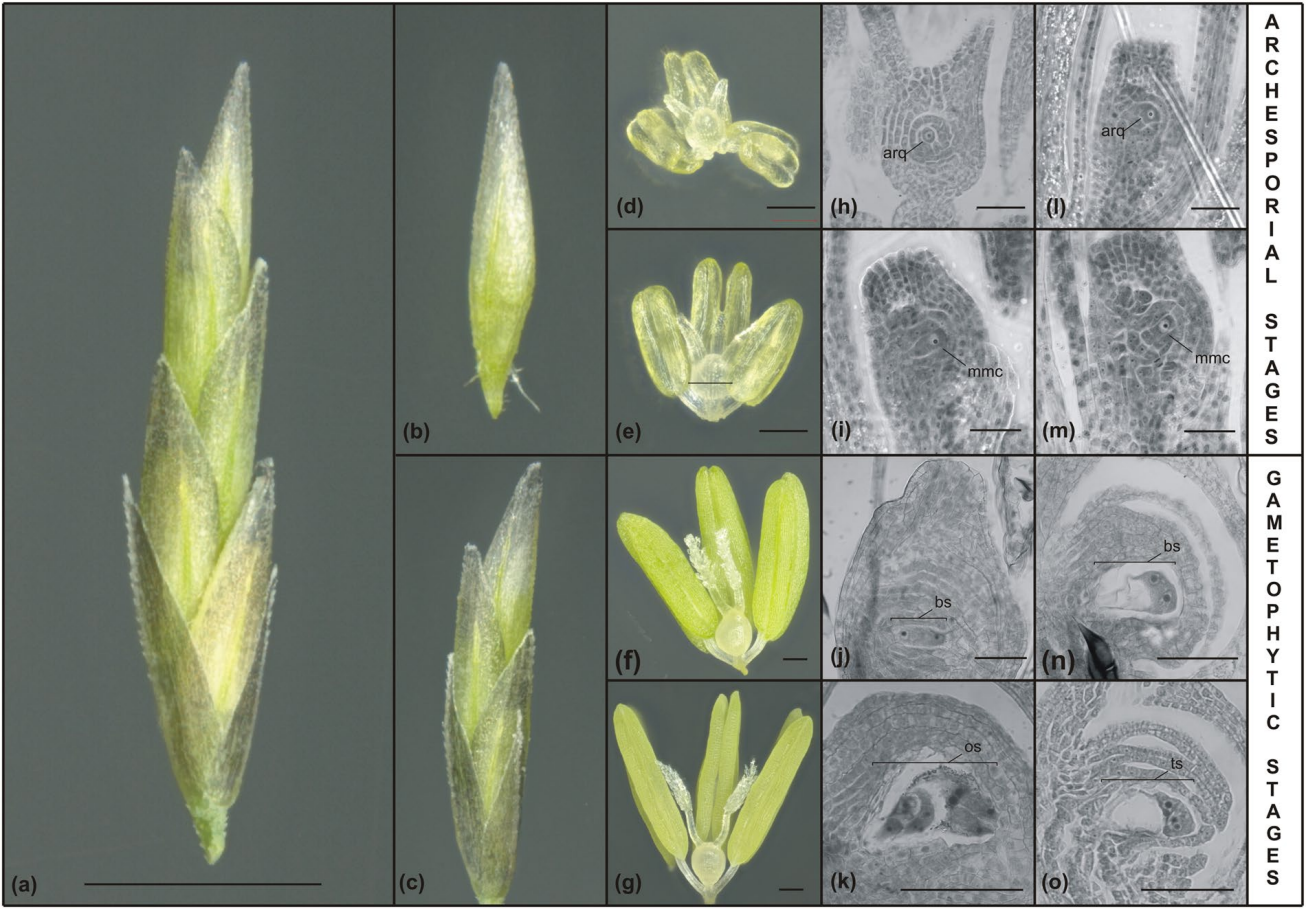
Reproductive calendar of sexual and apomictic individuals of *Eragrostis curvula*. (**a**) Whole spikelet with the anthecium containing all developmental stages. Scale bar: 1000 μm. (**b,c**) Stages used for total RNA extractions; (**b**) top of the spikelet with flowers containing ovules at archesporial and pre-meiotic stages, and (**c**) anthecium with pistils at gametophytic stages. (**d–g**) Ovaries and anthers at: (**d**) early archesporial stage, (**e**) late archesporial stage, (**f**) early gametophytic stage, and (**g**) late gametophytic stage. Scale bar: 100 μm. (**h–k**) Ovary sections from the sexual OTA genotype showing: (**h**) an archesporial cell, (**i**) a megaspore mother cell, (**j**) a binucleate embryo sac, and (**k**) a mature embryo sac with proliferating antipodal cells. Scale bar: 50 μm. (**l–o**) Ovary sections from Tanganyika apomicts showing embryo sac developmental course: (**l**) an archesporial cell, (**m**) a megaspore mother cell, (**n**) a binucleate embryo sac, and (**o**) a tetranucleate mature embryo sac. Scale bar: 50 μm. arq: arquespore, mmc: megaspore mother cell, bs: binucleate stage, oc: octonucleate stage, ts: tetranucleate stage.

**Figure 3:**
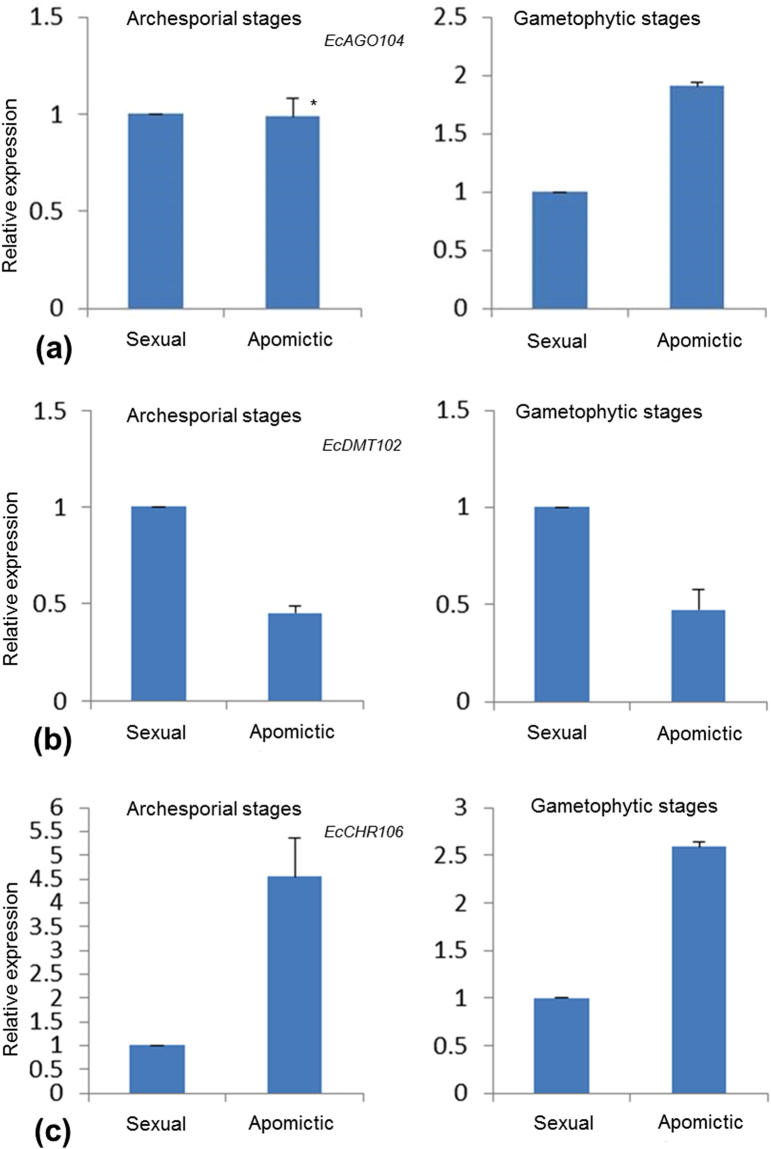
Quantitative RT-PCR of three RdDM genes during sexual and apomictic reproduction in *E*. *curvula*. Analyses were done using mRNA samples obtained from ovaries containing ovules at sporogenesis (archesporial stages) and megagametogenesis (gametophytic stages), Histograms show fold changes of expression levels for selected genes relative to control genes and error bars indicate the standard deviation for sample replicates. Differences between mean values were evaluated using Student’s t-tests with paired samples (P < 0.05). *Samples that were not validated

We first compared the expression profiles of *EcAGO104*, *EcDMT102* and *EcCHR106* using the 454-sequencing data derived from the sexual and apomictic libraries^17^. Differential expression analysis using the EdgeR package detected a significant difference only for *EcAGO104* that showed higher expression in apomictic plants compared to sexual counterparts (p value = 1.17E-05). These results are discordant with previous reports indicating downregulation for all three genes in apomict plants^15,16^. However, since our reference transcriptome was generated using a mix of all reproductive developmental stages, it is possible that we could not detect more subtle transcriptional differences using this approach. Therefore, we performed quantitative RT-PCR using mRNAs extracted from flowers at both archesporial and gametophytic stages to compare transcript abundances between sexual and apomictic plants (Fig. 2). No significant difference in expression was observed for *EcAGO104* between OTA-S and Tanganyika samples during archesporial stages; however Tanganyika flowers expressed significantly more *EcAGO104* than OTA-S flowers during gametogenesis (Fig. 3a). Furthermore, we detected higher expression levels of *EcDMT102* in sexual plants across all developmental stages (Fig. 3b), whereas *EcCHR106* transcripts were overrepresented in apomictic plants across both archesporial and gametophytic stages (Fig. 3c).

Thus, although expression patterns can differ from those observed in other species (i. e. *AGO104* and *CHR106*), we show here that the expression level of the selected candidate genes varies between apomictic and sexually reproducing plants, at least in specific developmental stages.

According to Olmedo-Monfil *et al*.^11^ most of AGO9-associated sRNAs are 24 nucleotides in length sRNAs and most 24-nucleotide sequences derived from TEs belonging to distinct families of retrotransposons: Gypsy (23%) Athila (9.3%), CACTA (5.5%), and less frequently LINE or Mutator.

Thus, we mined the *E*. *curvula* reference transcriptome of Garbus *et al*. (manuscript under revision) and identified 267 putative LTR retrotransposons, including known targets of AGO9 (i.e. Athila retrotransposons^20^). Out of these, 63 were differentially represented between sexual and apomictic plants, but the differences were not conclusive. On the other hand, in a previous report on repetitive sequences expressed in cDNA libraries obtained from *Eragrostis* ^21^, we showed differential expression for several members of the Gypsy retrotransposons family between inflorescences of sexual and apomictic genotypes. As already mentioned, our reference transcriptome covers all developmental stages. Since *AGO9* activity was shown necessary to silence TEs in female gametes and their accessory cells^11^, this might provide an explanation for these observations. Alternatively, we show here that rather than downregulated, *AGO9/AGO104* in *Eragrostis* apomicts is expressed in a different domain than in sexual plants. Therefore, such expression pattern could impact TE regulation in a different way that that reported for *AGO9* mutant plants of *Arabidopsis*.

### *In situ* hybridization analyses

**Figure 4:**
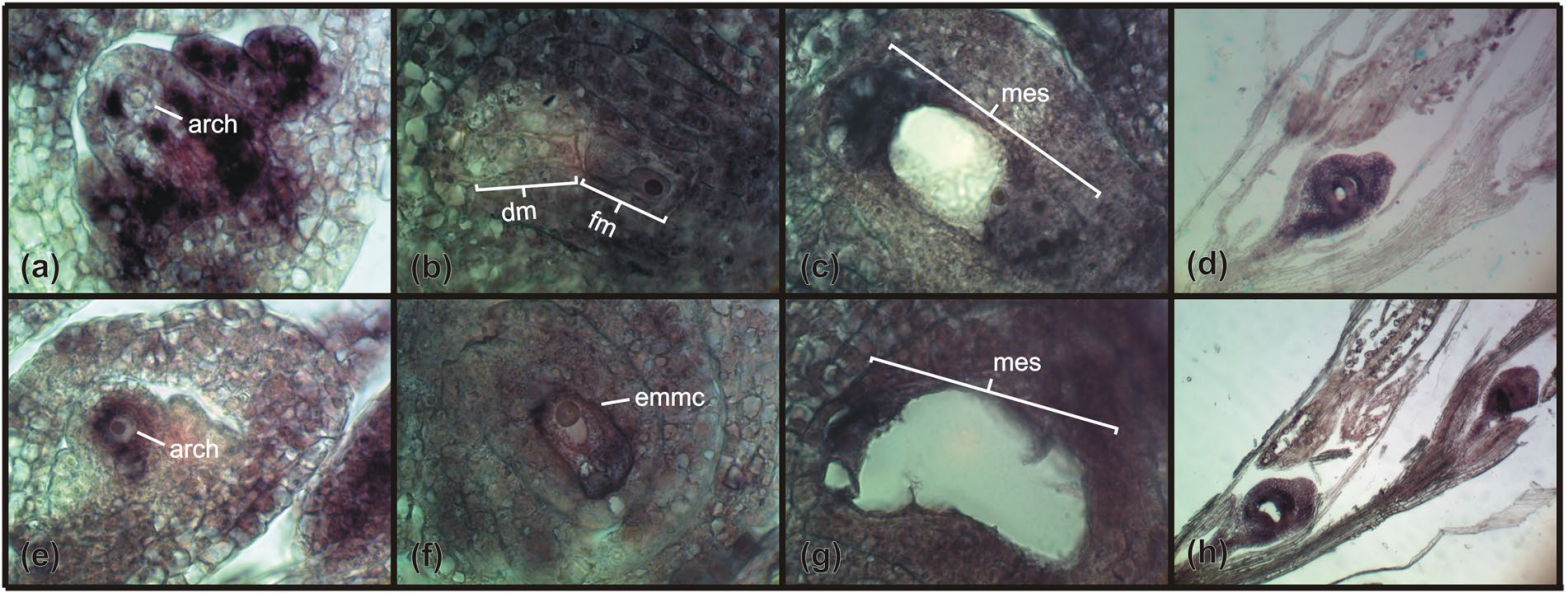
mRNA *in situ* hybridization of *EcAGO104* during sexual and apomictic reproduction in *E. curvula*. (**a–d**) Ovaries from the sexual genotype hybridized with an *EcAGO104* antisense probe showing: (**a**) an ovule primordium with strong, patchy signal in the nucellus and integuments and no signal in the archesporial cell; (**b**) signal restricted to the functional megaspore in a tetrad; (**c**) a mature embryo sac with all cells positive and no signal in the surrounding nucellar cells, and; (**d**) a general view of reproductive organs. (**e–h**) Ovaries from the apomictic genotype hybridized with an *EcAGO104* antisense probe showing: (**e**) an apomictic ovule primordium with a strong signal detected in the archesporial cell only; (**f**) an elongated, unreduced MMC; (**g**) a mature embryo sac, and; (**h**) a general view of reproductive organs. Arch: archespore, dm: degenerated megaspores, fm: functional megaspore, mes: mature embryo sac, emmc: elongated megaspore mother cell.

**Figure 5:**
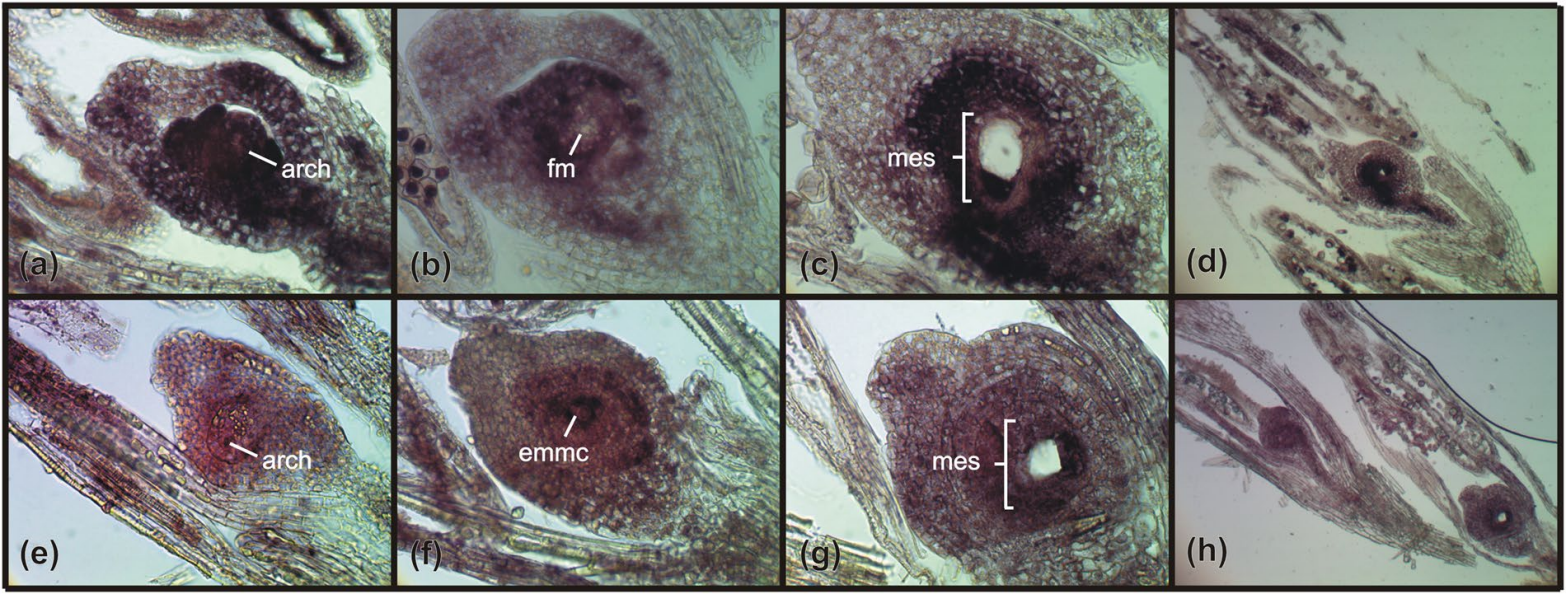
mRNA *in situ* hybridization of *EcDMT102* during sexual and apomictic reproduction in *E. curvula*. (**a**–**d**) Ovaries from the sexual genotype hybridized with an *EcDMT102* antisense probe showing strong signals in the nucellus and reproductive cells throughout development. (**a**) Young ovule primodium containing an archespore. (**b,c**) Ovules containing young and mature embryo sacs. (**d**) general view of reproductive organs. (**e–h**) Ovaries from the apomictic genotype hybridized with an *EcDMT102* antisense probe showing weaker, but similarly distributed than in the sexual plants, signals. (**e**) Young ovule primodium containing an archespore. (**f**) Ovule containing an enlarged, unreduced MMC. (**g**) Ovules containing mature embryo sac. (**h**) General view of reproductive organs. arch: archespore, dm: degenerated megaspores, fm: functional megaspore, mes: mature embryo sac, emmc: elongated megaspore mother cell.

Since plants lacking *AGO9/AGO104* and *DMT102* have reproductive phenotypes mimicking apomixis in *Arabidopsis* and maize^11,13,14^, *in situ* hybridization was conducted to determine the cell-specific expression patterns for these two genes in *E*. *curvula* reproductive organs (Figs 4 and 5). *EcAGO104* is expressed at archesporial stages in ovules of both sexual and apomictic plants, but with contrasting distribution patterns. In sexual plants, strong but dispersed hybridization signals were observed in the nucellus and the integuments, with no signal in the archesporial cell (Fig. 4a) and the MMC. By contrast, apomictic plants produced weak signals around the archesporial cell/MMC and a strong signal within the archesporial cell throughout development (Fig. 4e,f), although in the later stages, the signal seemed to be restricted only to the enlarged, unreduced MMC. Interestingly, *EcAGO104* was first detected in the sexual germ line in functional surviving megaspores while micropylar megaspores produced no signal (Fig. 4b). Throughout female gametogenesis in both sexual and apomictic plants, a moderate expression of *EcAGO104* was observed in the integuments and all cell types within the embryo sac (Fig. 4c,g). Finally, a strong signal was observed during male sporogenesis and gametogenesis in both somatic (tapetum) and reproductive cells (pollen mother cells, microspores) in both sexual and apomictic plants (Fig. 4d,h) indicating that *EcAGO104* was differentially regulated during male and female reproductive developments between apomictic and sexual *E. curvula* plants.

*In situ* hybridization of *EcDMT102* produced stronger signals in the ovules of sexual plants than apomictic plants across all female and male developmental stages (Fig. 5a–h), a result in agreement with both 454 sequencing and RT-qPCR analyses. Nevertheless, both the spatial and temporal expression patterns remained similar between genotypes. In the early stages, while both the nucellus and integuments stained strongly, the signal was either weak or absent in the reproductive cells, i.e. the sexual MMC (Fig. 5a) and both the early and enlarged diplosporous MMC (Fig. 5e,f). During gametogenesis, expression was detected in nucellar cells and within the embryo sac, particularly at the micropylar end (Fig. 5b,c,g). Finally, the expression in the anthers followed a similar pattern, with stronger signal in sexual plants than in apomict plants (Fig. 5d,h).

Finally, hybridizations of ovules from both sexual and apomict plants collected at different developmental stages showed no signal for *EcAGO104* sense probes whereas a moderate signal was observed for *EcDMT102* (Supplemental Figure S1). This suggests antisense expression at a restrained level for *EcDMT102*. This phenomenon has been commonly observed for other apomixis-related candidates complexes, e.g. *SOMATIC EMBRYOGENESIS RECEPTOR KINASE* ^22^ and *ORIGIN RECOGNITION COMPLEX3* ^23^, and it has been proposed to play a suppression role mediated by the formation sense-antisense mRNA complexes in apomict plants^22,23^.

## Discussion

Here, we report expression analyses in the ovules of apomictic and sexual *E. curvula* individuals for three genes involved in DNA methylation pathways. Our results agree with previous findings in model plant species suggesting that RdDM misregulation in the ovule may promote apomictic-like phenotypes^11–14^; however, published expression data has also highlighted both similarities and differences between different diplosporous apomicts for the genes examined so far, i.e. two *DMT*s and *CHR106/DDM1* ^14^. In *B. holboellii* and *T. dactyloides*, *CMT3/DMT102/DMT105* was downregulated in apomictic plants^14^, in agreement with the results reported here for *E. curvula*. Contrastingly, *CHR106/DDM1* was downregulated in *T. dactyloides* apomictic plants^14^, and showed no difference in *B. holboellii* apomictic and sexual plants, while we report here upregulation in *E. curvula* apomicts. In the present study, both quantitative and tissue-specific expression differences were detected for two genes (*EcAGO104* and *EcDMT102*), changes which had already been associated with apomixis-like phenotypes in *Arabidopsis* and maize^11,13,14^.

AGO9/AGO104 proteins are specifically expressed in ovules of plants and are essential to specify cell fate, a function achieved by silencing heterochromatic sequences and, probably, genes^11,13^. However, mutant characterization in *Arabidopsis* and maize has revealed different functions during female reproduction, respectively repression of reproductive cell fate in the ovule and repression of somatic cell fate in the archespore. Therefore, loss-of-function in these two species generate different apomixis-like phenotypes reminiscent of apospory (*Arabidopsis*) and diplospory (maize). Our *in-situ* hybridization observations for *EcAGO104* first suggest a similar function to that of maize AGO104 proteins. We detected signals in the female reproductive cell lineage from functional megaspores to mature embryo sacs in sexual plants and throughout all reproductive development in apomicts, including archesporial cells. This may indicates that ectopic *EcAGO104* expression in the archespore could prevent entry into meiosis and promote gametophytic development. The weaker expression of *EcAGO104* in the nucellus of apomictic plants before and during sporogenesis relative to that in sexually reproducing plants is an intriguing observation. Whether the phenomenon is required for triggering ectopic expression in the archespore remains unclear. One explanation, in agreement with the hypothesis for a non-cell autonomous, silencing signal spreading from sporophytic cells into reproductive cells during sporogenesis^3^, is to postulate an autoregulatory loop controlling *EcAG0104* expression during sexual sporogenesis depending on mobile small RNAs generated in surrounding nucellar cells. Therefore, although our data also support a silencing pathway involving AGO9/AGO104 operating in the nucellus of sexual plants and promoting meiosis, more analyses in both apomictic and sexual plants are required to decipher the mechanisms governing *AGO9/AG0104* expression and to assess AGO9/AGO104 function in plant ovules.

DMT102 is required for cytosine methylation at CNG sites, and it is likely involved in a maintenance function^24,25^. In maize, *DMT102* expression domain encompasses the reproductive cell and a few layers of nucellar cells^14^. This domain is conserved during early gametogenesis, while in mature ovules *DMT102* expression becomes confined to the chalazal region of the embryo sac^14^. Similarly, in sexual *E. curvula* individuals, a strong signal was observed in the nucellus during early premeiosis; however, after gametogenesis, expression was detectable throughout the embryo sac (not only at the chalazal end).

In *dmt102* maize mutants, no apparent morphological nor reproductive phenotype, other than a high proportion of abnormally large pollen grains, was detected, and *dmt102::Mu* alleles are normally inherited both maternally and paternally^14^. In *E. curvula* diplosporous plants, we observed a significant level of *EcDMT102* expression in the anthers, which might explain the absence of non-reduced pollen^26^. Maize *dmt102* homozygous mutant lines produce nearly full seed sets, indicating that most, if not all, female gametes are meiotically derived; however, at late gametophytic stages, large structures develop at the chalazal pole of the ovule, presumably after abnormal growth of antipodal cells^14^. These large chalazal cells remained arrested until the multicellular antipodal structure degenerates, suggesting that DMT102 might be involved in the maintenance of antipodal cell fate during late gametogenesis^14^. Interestingly, a downregulation of *EcDMT102* expression was observed in the nucellus of diplosporous *E. curvula* plants. These plants form embryo sacs lacking antipodal cells; therefore, it could be hypothesized that the natural downregulation of *EcDMT102* in this species might be promoting the establishment of gametophytic specificities characteristic of apomictic individuals (ie, four-celled embryo sac), which differ from the canonical seven-celled embryo sacs observed in sexual individuals.

Contrary to observations indicating either down-regulation in maize-*Tripsacum* apomicts or steady-state expression in *Boechera* ^14^, we found evidence for increased expression in *Eragrostis* apomicts of *EcCHR106*, an ortholog of the well-characterized *Arabidopsis DDM1* gene. *AtDDM1* encodes a nucleosome remodeling ATPase^27^, allowing DNA methyltransferases to access H1-containing heterochromatin^28^. In *Arabidopsis*, DDM1 and RdDM are both active in all DNA methylation contexts to silence TE, but with different efficiencies depending on chromatin organization. DDM1 is less efficient for short TEs, usually found nearby genes (ie, euchromatic regions) and, therefore, silencing relies more on RdDM. Oppositely, silencing in heterochromatic regions is more effective through DDM1 than RdDM^28^. Our data indicate that DDM1 function is enhanced, or at least preserved, throughout ovule development in *Eragrostis* apomicts while RdDM is inactivated. This suggests that chromatin states nearby and within genes could be more permissive in apomict ovules and, therefore, supports the notion that RdDM is involved in the establishment of gene expression patterns governing the switch from sexuality to diplosporous apomixis in plants. Recent progress in understanding DNA methylation patterns in plants has revealed differences in mechanisms and functions for both canonical and non-canonical RdDM pathways across species^29,30^. These specificities might have sustained the emergence of diversified reproductive behaviors, including the differences observed among apomictic developments.

## Materials and Methods

### Biological materials and resources

Two tetraploid (2n = 4x = 40) accessions of weeping lovegrass provided by the United States Department of Agriculture (USDA) were used: the apomictic Tanganyika (PI234217) and the sexual OTA-S (PI574506) genotypes. Plants were grown in a glasshouse at 25 °C under natural light conditions. For both genotypes, a reproductive calendar was compiled based on anatomical (spikelets size and morphology) and histological observations. This calendar encompasses four developmental stages from early archespores to mature female gametophytes (Fig. 2).

### FLX Roche 454 sequencing

Spikelets with basal flowers at the beginning of anthesis, representing all developmental stages, were collected and total RNA was extracted using a NucleoSpin® miRNA kit (Machery-Nagel, Düren, Germany). Each genotype was represented by two biological replicates. The samples were sequenced using the 454 GS FLX + Roche method at INDEAR (Instituto de Agrobiotecnología de Rosario, Santa Fe, Argentina). Raw reads were deposited in the Sequence Reads Archive (SRA) database at NCBI as BioProject 358210 “Floral transcriptome of sexual and apomictic *Eragrostis curvula*” including Biosamples SAMN06167423 (reads from OTA-S) and SAMN06167424 (reads from Tanganyika). Curated raw sequences were assembled using Newbler Assembler software v2.6 (Roche, Indianapolis, IN, USA).

Differential expression between two conditions was analyzed using the EdgeR package^31^ and P-values adjusted using the Benjamini and Hochberg method^32^ to control the false discovery rate. A corrected P-value of 0.01 and log2 (fold-change) of 1 were used as thresholds for significant differential expression.

### Phylogenetic and sequence analyses

*Arabidopsis* and maize protein sequences for *AtAGO9/ZmAGO104*, *AtDDM1/*Zm*CHR106*, and *AtCMT3/ZmDMT102* were retrieved, respectively, from The *Arabidopsis* Information Resource (TAIR) and the Maize Genetic and Genomic Database (MaizeGDB), and used to query the 454 RNA libraries using BLASTP. Gene families were aligned using a BLOSUM30 matrix^33^ and ClustalW in pairwise alignments (open gap penalty: 10; gap extend penalty: 0.1) and multiple alignments (gap extend penalty: 0.2; delay divergent setting: 30%) were generated. A phylogenetic reconstruction was conducted using a neighbor-joining method in MEGA v6.0^34^. Unrooted consensus trees were obtained from 1000 bootstrap replicates.

### RT-qPCR experiments

Flowers from OTA-S (sexual plant) and Tanganyika (apomictic plant) were collected and divided in both archesporial and gametophytic stages (Fig. 2). Total RNA was extracted as described above. The RNA was reverse-transcribed using the ImProm-II™ Reverse Transcription System (Promega, Madison, WI, USA). Real-time PCR reactions were conducted as previously^35^, using primers designed with the SciTools software (http://www.idtdna.com) from Integrated DNA Technologies (Coralville, IA, USA) (Supplementary Table S1). *UBIQUITIN CONJUGATING ENZYME* (*UBICE*) and *GLUCOSE-6-PHOSPHATE DEHYDROGENASE* (*GPDH*) were used as reference genes, after validating their steady-state expression patterns throughout reproductive development in the plants using the gNorm software^36^ (data not shown). Non-template controls were incorporated into all assays. For each sample, two biological replicates were processed in triplicate. Amplifications were performed in a Rotor-Gene 6000 (Corbett Life Sciences) using the following conditions: 2 min at 94 °C, followed by 40 cycles of 15 s at 95 °C, 20 s at 55 °C, and 30 s at 72 °C. A melting curve was produced at the end of each reaction to check amplicon specificity. Amplification efficiency was calculated with Rotor-Gene 6000 Series Software 1.7. Relative gene expression was assessed using the 2^−ΛΛCT^ method^37^. Differences between mean values were evaluated by Student’s t-tests. P values < 0.05 were considered significant.

### *In situ* hybridization experiments

Sense and antisense DIG-labelled RNA probes were produced for the *EcAGO104* and *EcDMT102* genes. Hybridizations were performed for the ovaries of plants at different developmental stages, following the protocol previously described by Laspina *et al*.^38^ with minor modifications. Briefly, inflorescences at pre-anthesis were fixed in 4% paraformaldehyde/0.25% glutaraldehyde in 0.01 M phosphate buffer pH 7.2, dehydrated in an ethanol–xylol series and embedded in paraffin. Spikelets were cut into 7–10 μm thin sections and placed onto slides treated with 100 μg/ml poly-l-lysine. Paraffin was removed using an ethanol-xylol series. Plasmids containing the selected clones were linearized using the restriction enzymes NcoI or SalI (Promega). The amplicons 577-nt and 555-nt long originated from *EcAGO104* and *EcDMT102* were used as probes in hybridization analysis (Supplementary Table S1). Probes were labeled using the Roche Dig RNA Labeling kit, following the manufacturer’s instructions. The probes were hydrolyzed to 150–200 bp fragments. Pre-hybridization was carried out in 0.05 M Tris–HCl pH 7.5 buffer containing 1 μg/mL proteinase K in a humid chamber at 37 °C for 10 min. Hybridization was carried out overnight in a humid chamber at 37 °C, in 10 mM Tris–HCl pH 7.5 buffer containing 300 mM NaCl, 50% formamide (deionized), 1 mM EDTA pH 8, 1x Denhardt, 10% dextransulphate, 600 ng/ml total RNA and 60 ng of the corresponding probe. Detection was performed following the Roche Dig Detection kit instructions, using anti-DIG AP and NBT/BCIP. Three independent experiments, each involving at least 20 spikelets, were conducted per genotype.

## Electronic supplementary material

Figure S1 and Table S1
