# Supplementary material for: Temporal and spatial expression of genes involved in DNA methylation during reproductive development of sexual and apomictic Eragrostis curvula
Source: Sci Rep. 2017 Nov 8;7:15092. doi: 10.1038/s41598-017-14898-5 (PMC5678148; doi:10.1038/s41598-017-14898-5)
Supplement: Supplementary file 1 — Figure S1 and Table S1 [file 41598_2017_14898_MOESM1_ESM.pdf]

Temporal and spatial expression of genes involved in DNA methylation during reproductive development of sexual and apomictic Eragrostis curvula

Selva JP, Siena L, Rodrigo JM, Garbus I, Zappacosta D, Romero JR, Ortiz JPA, Pessino SC, Leblanc O* & Echenique V*

Supplementary Figure S1. mRNA in situ hybridization using sense probes in *E. curvula* ovaries. (a, b) EcAGO104 sense probe detected no signal in ovaries of both sexual and apomictic plants containing, respectively, an archesporial cell (a) an elongated megaspore mother cell (b). (c-d) EcDMT102 sense probe produced some levels of signal, usually in the tissues surrounding the reproductive cells, during sexual and apomictic reproduction: (c) Ovary containing a megaspore mother cell in a sexual plant. (d) Ovary containing a mature embryo sac in an apomictic plant. arch: archespore, emmc: elongated megaspore mother cell, mes: mature embryo sac, mmc: megaspore mother cell.

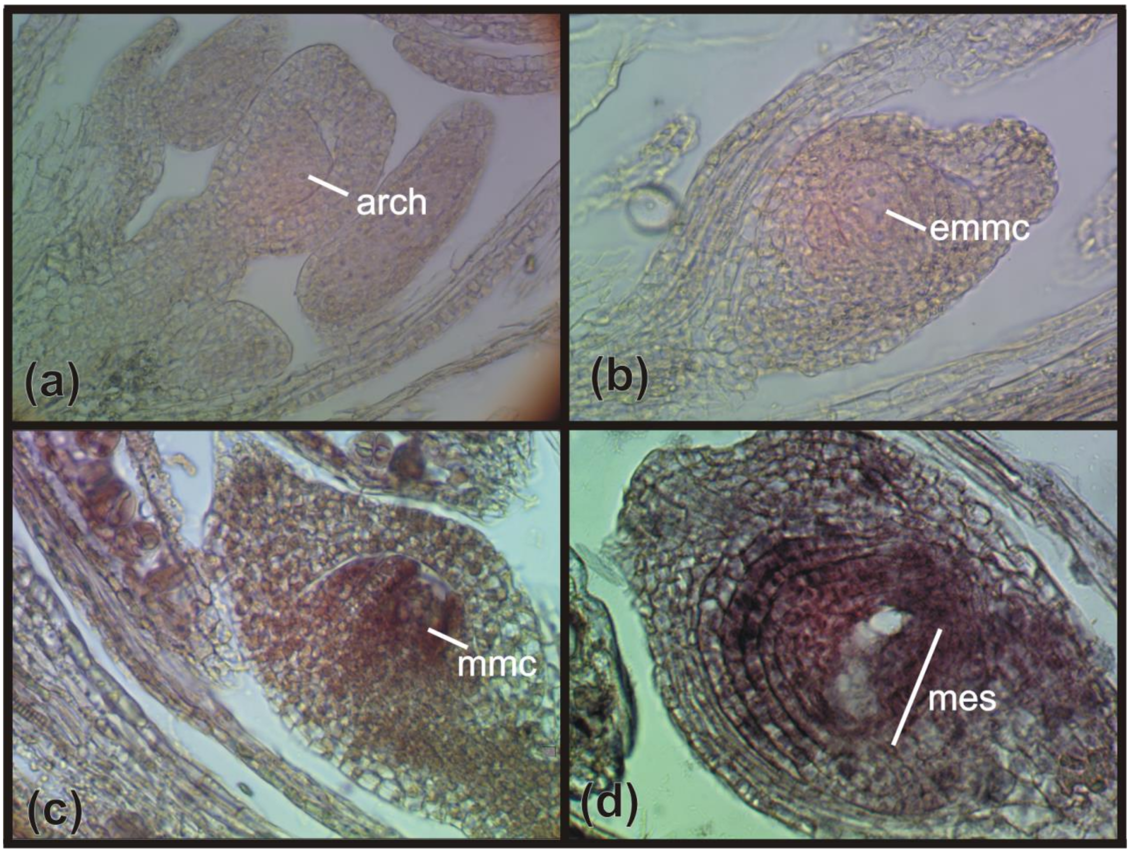

Table S1: Primer pairs used for qRT-PCR analysis and amplicons used for *in situ* hybridization.

Primer name	sequence 5' - 3'	Isotig	Amplicon size
EcAGO104qPCR FWD	CACTGTAGTTCTGGAGGATGTG	isotig33988	114
EcAGO104qPCR REV	GTCTGATATGGCCTCCTCATTC		
EcAGO104qPCR FWD	CACTGTAGTTCTGGAGGATGTG		577
EcAGO104 REV	CTTCCTGTCACTCAGACCAATAA		
EcDMT102qPCR FWD	CCAGCAGTGCAGCATCTAATA	isotig25116	135
EcDMT102qPCR REV	CACATCCAGAGTACAGGTCAAG		
EcDMT102qPCR FWD	CCAGCAGTGCAGCATCTAATA		555
EcDMT102 REV	GGCAATCCCTAAGACCATCAA		
EcCHR106 qPCR FWD	CGGCCTCATCCAAGACATTAG	isotig25194	112
EcCHR106 qPCR REV	GAGGGACGGATCATCAAGAAAG		
